# Supplementary material for: A yeast phenomic model for the influence of Warburg metabolism on genetic buffering of doxorubicin
Source: Cancer Metab. 2019 Oct 23;7:9. doi: 10.1186/s40170-019-0201-3 (PMC6806529; doi:10.1186/s40170-019-0201-3)
Supplement: Supplementary file 9 — Additional file 9. GO term-specific heatmaps for REMc/GTF-enriched clusters. GO term-specific heatmaps for significant GO process terms were generated as described in methods and Figs. 3 and 4. Any related child terms are presented in subsequent pages of the parent file name. GO terms with more than 100 children, with 2 or fewer genes annotated to the term, or a file size over 300KB are not shown. All heatmaps are generated with the same layout (see Figs. 3 and 4). [file 40170_2019_201_MOESM9_ESM.bz2 › Additional_File9_GOTermHeatmaps/Additional_File9_GOTermHeatmap/regulation_of_intracellular_pH.pdf]

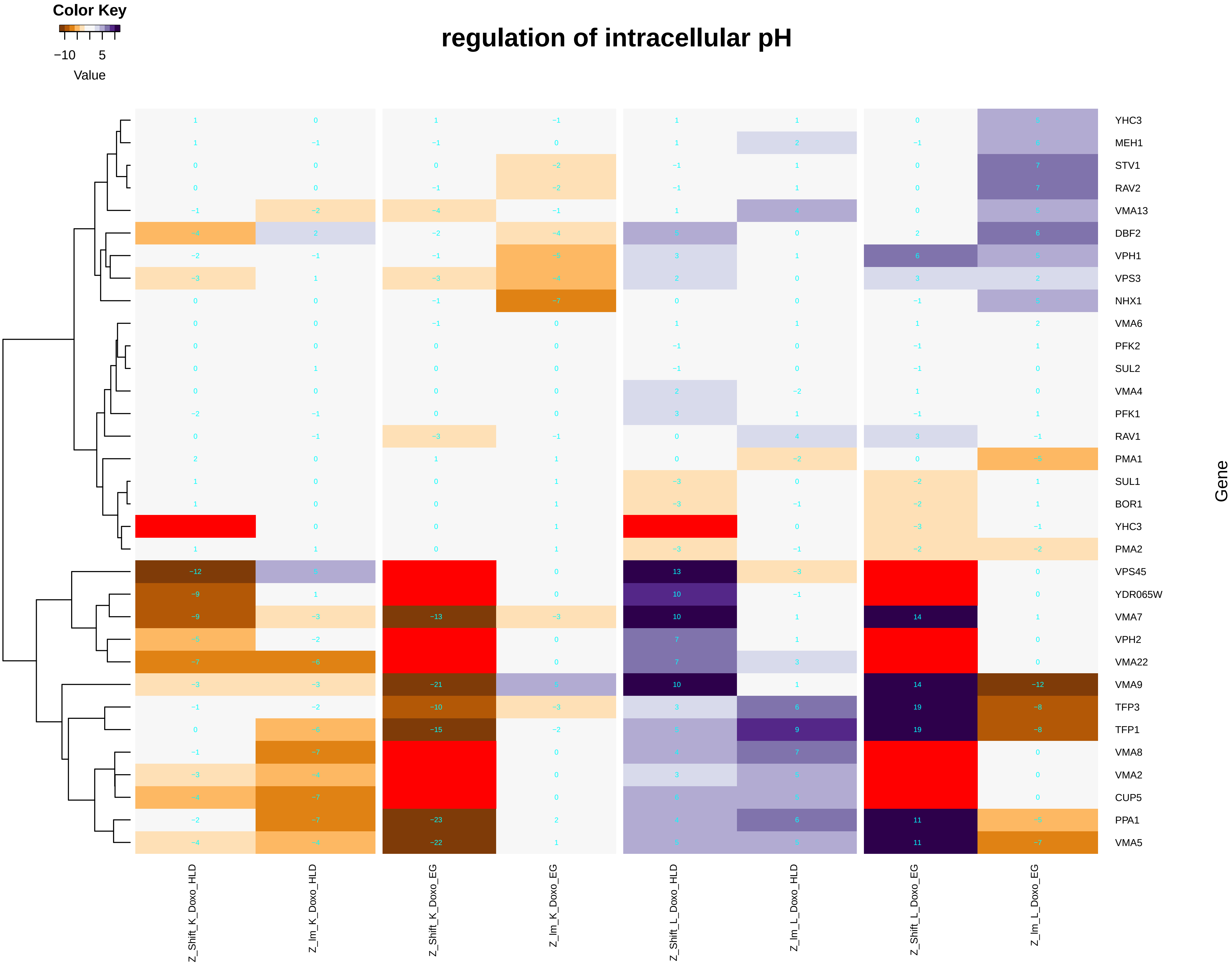
Color Key
−10
5
Value
regulation of intracellular pH
YHC3
MEH1
STV1
RAV2
VMA13
DBF2
VPH1
VPS3
NHX1
VMA6
PFK2
SUL2
VMA4
PFK1
RAV1
PMA1
SUL1
BOR1
YHC3
PMA2
VPS45
YDR065W
VMA7
VPH2
VMA22
VMA9
TFP3
TFP1
VMA8
VMA2
CUP5
PPA1
VMA5
Gene
Z_Shift_K_Doxo_HLD
Z_Im_K_Doxo_HLD
Z_Shift_K_Doxo_EG
Z_Im_K_Doxo_EG
Z_Shift_L_Doxo_HLD
Z_Im_L_Doxo_HLD
Z_Shift_L_Doxo_EG
Z_Im_L_Doxo_EG

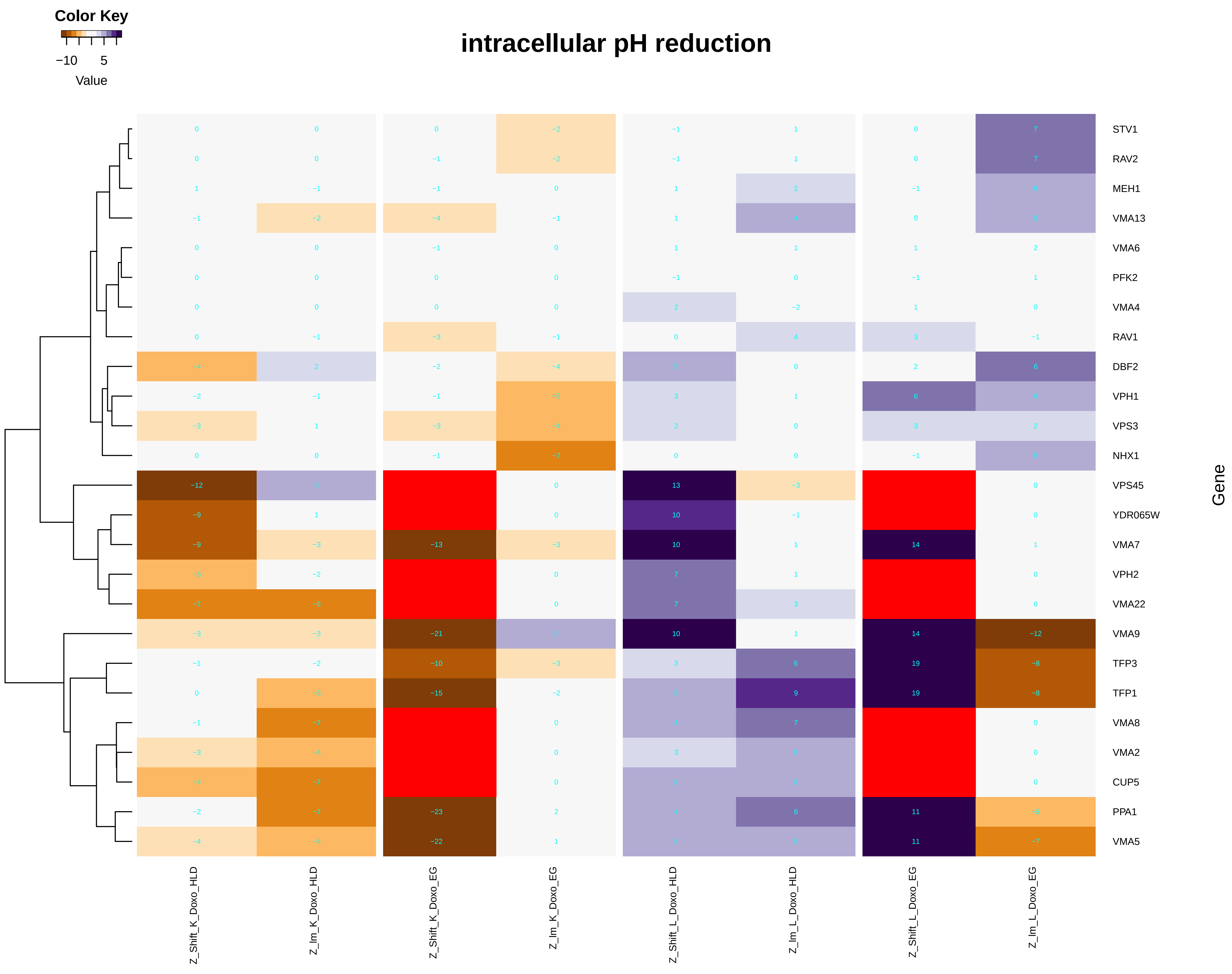

Color Key
−10
5
Value
intracellular pH reduction
STV1
RAV2
MEH1
VMA13
VMA6
PFK2
VMA4
RAV1
DBF2
VPH1
VPS3
NHX1
VPS45
YDR065W
VMA7
VPH2
VMA22
VMA9
TFP3
TFP1
VMA8
VMA2
CUP5
PPA1
VMA5
Gene
Z_Shift_K_Doxo_HLD
Z_Im_K_Doxo_HLD
Z_Shift_K_Doxo_EG
Z_Im_K_Doxo_EG
Z_Shift_L_Doxo_HLD
Z_Im_L_Doxo_HLD
Z_Shift_L_Doxo_EG
Z_Im_L_Doxo_EG

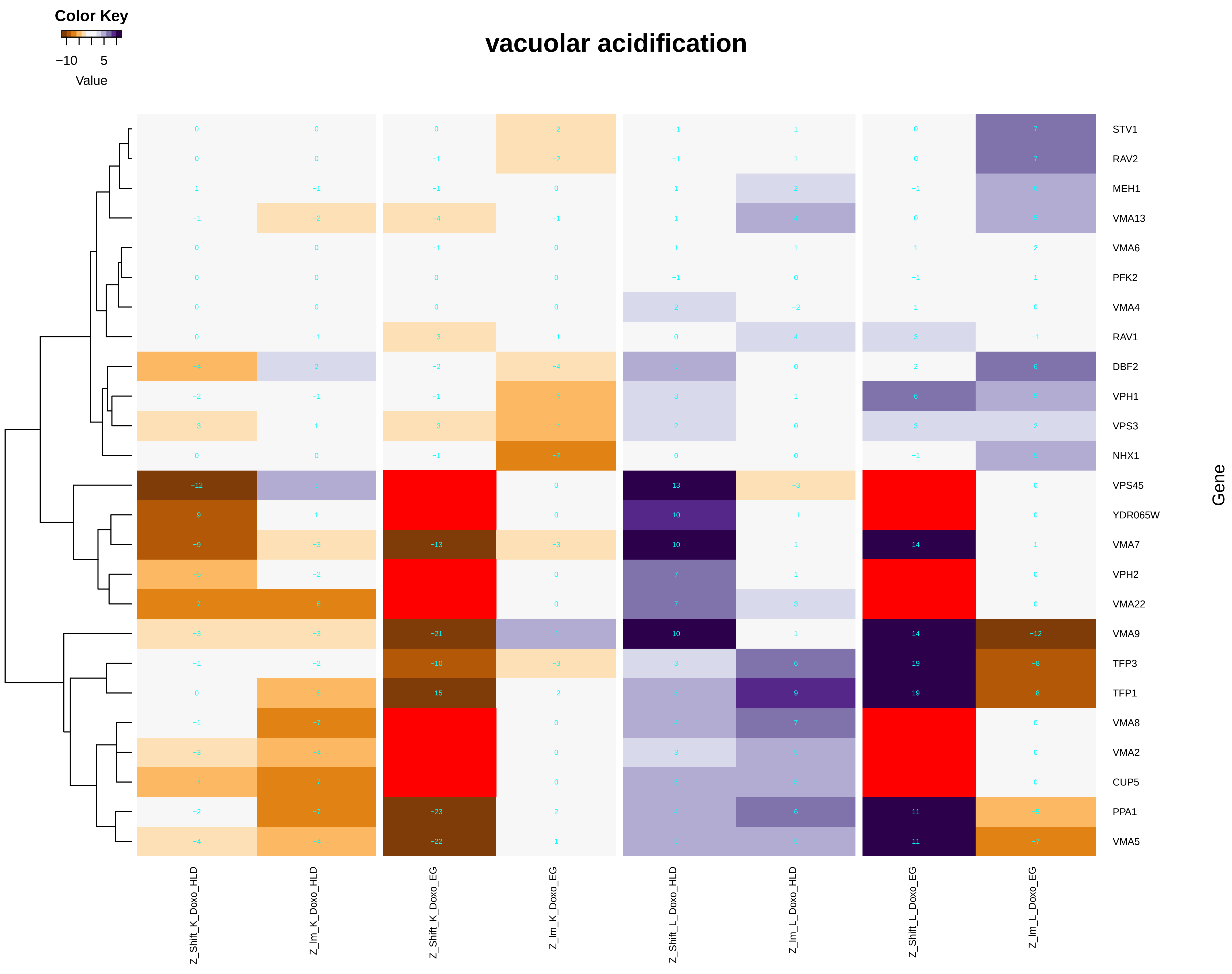
Color Key
−10
5
Value
vacuolar acidification
STV1
RAV2
MEH1
VMA13
VMA6
PFK2
VMA4
RAV1
DBF2
VPH1
VPS3
NHX1
VPS45
YDR065W
VMA7
VPH2
VMA22
VMA9
TFP3
TFP1
VMA8
VMA2
CUP5
PPA1
VMA5
Gene
Z_Shift_K_Doxo_HLD
Z_Im_K_Doxo_HLD
Z_Shift_K_Doxo_EG
Z_Im_K_Doxo_EG
Z_Shift_L_Doxo_HLD
Z_Im_L_Doxo_HLD
Z_Shift_L_Doxo_EG
Z_Im_L_Doxo_EG
